# Electroacupuncture alleviates ventilator-induced lung injury in mice by inhibiting the TLR4/NF-κB signaling pathway

**DOI:** 10.1186/s12871-024-02408-w

**Published:** 2024-01-23

**Authors:** Shuang Zhang, Shuji Li, Qingmei Liu, Daneng Wei, Liping Huang, Hong Yin, Mingliang Yi

**Affiliations:** 1 Department of Anesthesiology, The Second Clinical Medical College, Geriatric Diseases Institute of Chengdu/Cancer Prevention and Treatment Institute of Chengdu, Chengdu Fifth People’s Hospital, Affiliated Fifth People’s Hospital of Chengdu University of Traditional Chinese Medicine), Chengdu, 611137 Sichuan Province China; 2 Department of Anesthesiology, North Sichuan Medical College, Chengdu Fifth People’s Hospital, Chengdu, 611137 Sichuan Province China

**Keywords:** Electroacupuncture, Mechanical ventilation, Lung injury, TLR4/NF- κB signaling

## Abstract

**Objective:**

This study was aimed to explore the protective effect of electroacupuncture (EA) pretreatment at Zusanli point (ST36) on ventilation-induced lung injury (VILI) and its potential anti-inflammatory mechanism.

**Methods:**

High tidal volume ventilation was used to induce the VILI in mice, and EA pretreatment at ST36 was given for 7 consecutive days. The wet/dry ratio and pathological injury score of lung tissue, and total protein content of pulmonary alveolar lavage fluid (BALF) were detected after 4 h of mechanical ventilation (MV). Meanwhile, the expressions of TLR4 and NF- κB in lung tissue were evaluated by Western Blot, and the inflammatory factors in lung tissue were detected by ELISA.

**Results:**

After four hours of mechanical ventilation, mice with ventilator-induced lung injury showed significant increases in lung wet/dry ratio, tissue damage scores, and protein content in bronchoalveolar lavage fluid. Pro-inflammatory cytokines (IL-6, IL-1β, TNF-α) and TLR4/NF-κB expression levels in the lung were also markedly elevated (*P* < 0.05). Conversely, ST36 acupuncture point pre-treatment significantly reduced these parameters (*P* < 0.05).

**Conclusion:**

EA pretreatment at ST36 could alleviate the inflammatory response for VILI via inhibiting TLR4/NF- κB pathway.

## Background

Mechanical ventilation (MV) is not only a necessary means for general anesthesia patients to maintain their lives, but also an important measure for respiratory support treatment of critically ill patients, saving the lives of countless patients with severely impaired respiratory function. However, MV may also result to or aggravate lung injury as an injury factor [1]. Clinically, this MV-associated lung injury is known as ventilator-induced lung injury (VILI). It is generally believed that VILI is due to excessive alveolar tension caused by repeated closure and reexpansion of alveoli. Improper MV destroys alveolar epithelial cells and vascular endothelial cells, increases lung tissue permeability and leads to pulmonary edema and hypoxemia, thus resulting to VILI. VILI is characterized as diffuse alveolar-vascular membrane injury and increased permeability of the lungs, which mainly involves in barometric injury, volumetric injury, lung atrophy injury and biological injury. In clinical settings, the use of mechanical ventilators commonly leads to barotrauma, volutrauma, and atelectrauma, but the underlying mechanism of biological injury has not been clarified clearly [2, 3].

Toll-like receptors (TLRs) play an indispensable role in innate immune response, recognizing different pathogen-associated molecular patterns (PAMPs) and playing a key role in inflammation, immune cell regulation, survival and proliferation, as the first line to defend against pathogen invasion [4]. Toll-like receptors 4 (TLR4) is considered to be PAMPs sensors that induce the release of inflammatory chemokines and cytokines by activating the nuclear factor kappa-light chain enhancer in activated B cells (NF-κB) [5]. Previous studies have shown that TLR4/NF-κB signaling pathway is critical to the pathogenesis of ventilator-associated lung injury [6–8].

Series of studies have shown that weak electrical stimulation of nerves may beneficial for improving systemic inflammatory responses. Moreover, electroacupuncture (EA) stimulating at the ST36 acupoint on the hind limb of mice, could inhibit the inflammatory response of sepsis mice by using weak 0.5 mA current [9]. EA can also alleviate the acute lung injury caused by sepsis [10]. However, whether EA has a protective effect against the VILI remains unclear. Therefore, this study was aimed to clarify whether EA may mitigate VILI and identify the underlying molecular mechanisms.

## Methods

### Animals and experimental design

Experimental animals: SPF-grade male C57BL/6J mice, aged 8–12 weeks and weighing 22 ± 2 g, purchased from SPF (Beijing) Biotechnology Co., LTD., Animal Production license No. SCXK(Beijing)2019-0010. The mice were given adaptive feeding for 1 week, free to eat and drink, 12 h of light / 12 h of night. The temperature was 25 ± 2℃ and the humidity was 50 ± 5%. The experimental animals were completely randomly divided into the CTR group: the mice kept breathing autonomously after endotracheal intubation; VILI group: endotracheal intubation was performed after anesthesia, connected to ventilator, continuous mechanical ventilation for 4 h; SEA group: acupuncture pretreatment was performed 0.5 cm next to ST36 for 7d before mechanical ventilation for 4 h; EA group: EA at ST36 for 7d before mechanical ventilation for 4 h, with 8 animals in each group.

**Fig. 1:**
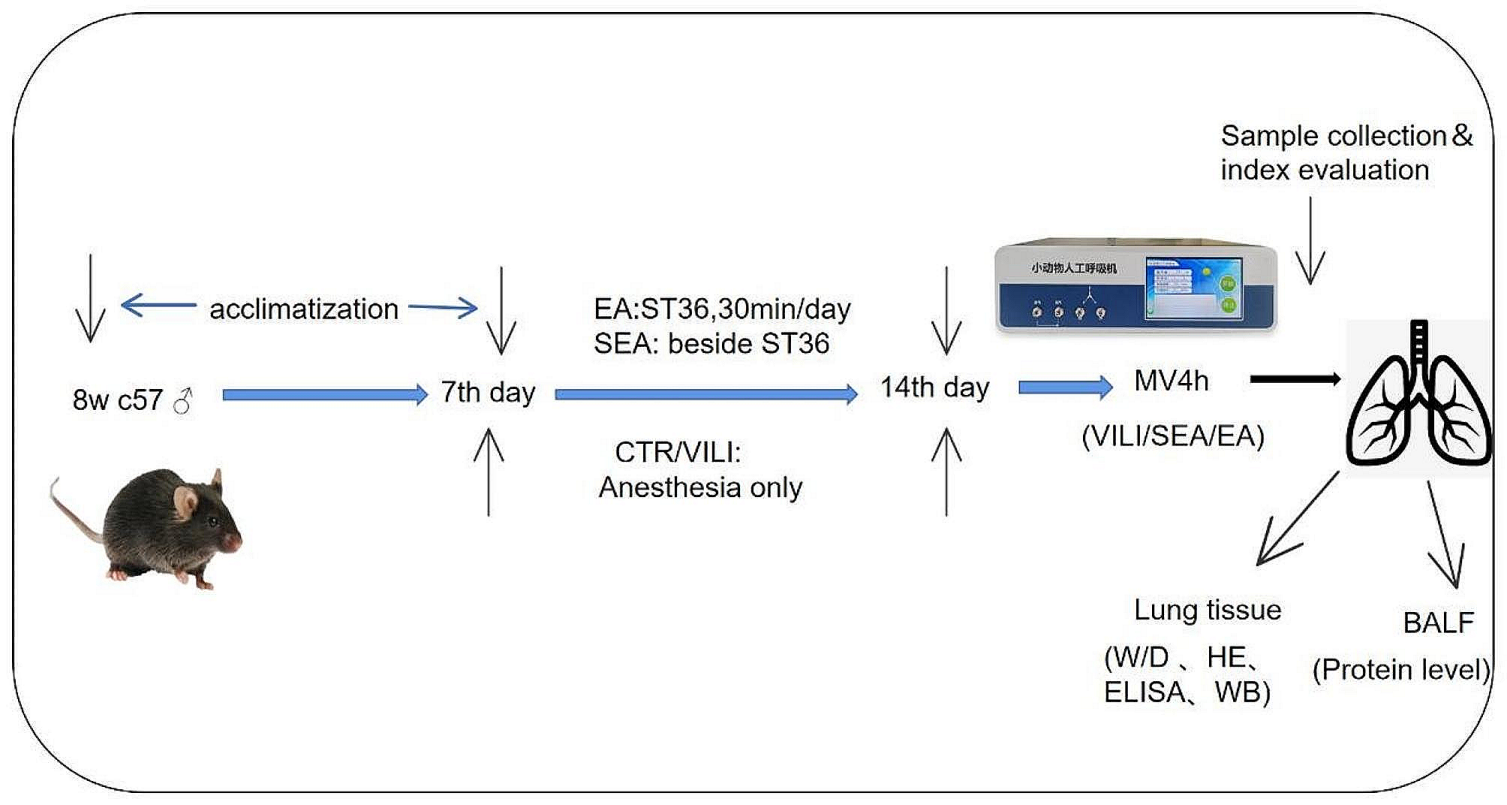
Schematic diagram illustrating the major steps of the experiment. (EA:electroacupuncture, SEA:shamelectroacupuncture, CTR:control, VILI:ventilation-induced lunginjury, MV:mechanicalventilation, W/D:wet/dryweight, HE:hematoxylineosinstaining, ELISA:enzyme-linkedimmunosorbentassay, WB:western blot, BALF: Bronchoalveolar lavage fluid.)

The experimental process is shown in Fig. 1. We used resource equation approach to calculate the sample size [11].During the experiment, the animals were disposed in accordance with the Guidelines on Treating Laboratory Animals Kindly issued by the Ministry of Science and Technology of the People’s Republic of China .

### Electroacupuncture method of ST36 in mice

Mice were anesthetized by intraperitoneal injection of 1% pentobarbital sodium (35 mg/kg) and electroacupuncture at ST36 acupoint on their hind limbs: two pairs of needles were inserted about 4 mm below the knee joint and about 2 mm outside the anterior tubercle of the tibia. The electric needle was inserted diagonally into the dermal tissue of the skin 3-4 mm.EA is carried out with a single-pole stainless steel needle (0.16 × 7 mm). The current range was set to 0.5-1 mA, the stimulation lasted for 30 min/d, the frequency was 2 Hz, and the left and right limbs were alternately controlled by the Korean electroacupuncture instrument, lasting for 7 days [9].

### Establishment of VILI

Mice were anesthetized with an intraperitoneal injection of 1% pentobarbital sodium at a dose of 35 mg/kg. Once anesthetized, the mice were placed on a tracheal intubation platform. A 22G catheter was inserted into the exposed glottis and connected to the ventilator to commence mechanical ventilation for 4 h. The ventilation parameters were set as follows: a tidal volume of 30 ml/kg, a respiratory rate of 100 breaths per minute, and an inspiration to expiration ratio of 1:2. The fraction of inspired oxygen (FiO2) was kept at 21% [12, 13].

After the end of ventilation, the mice were killed by intraperitoneal injection of pentobarbital sodium (50 mg/kg) under excessive anesthesia. Lung tissue and alveolar lavage fluid were collected for index detection.

### Determination of total protein in alveolar lavage fluid (BALF)

At the end of perfusion, alveolar lavage was performed through endotracheal intubation. The specific method was repeated perfusion with 0.3 ml PBS at 4℃ for 3 times, retention for 3 s and recovery. BALF recovered from 3 times of lavage was collected for mixed measurement with the alveolar lavage fluid retrieval efficiency of 90%. The supernatant of the collected BALF was taken after 400 RPM/separator at 4℃ for 5 min and immediately placed in the refrigerator at -80℃, and then taken out to be measured. BCA kit (Nanjing Nuoweizan Biotechnology Co.,LTD.,Nanjing) was used to detect the protein concentration in BALF.

### Measurement of lung tissue water content

The apical perfusion was performed with 4℃ PBS 5 ml, then superior lobe and middle lobe of right lung was taken weighed as wet weight by an electronic balance (accuracy 1 × 10^-4^). After weighing, the lung tissue was baked in a 60 °C oven for 24 h. It was weighed periodically until the weight remained constant, signifying the dry weight of the lung tissue. The change of water content of lung tissue was expressed by wet/dry weight.

### Lung injury score

After perfusion, a 2 mm thin slice of the left lung lobe was placed in a specimen clamp and immediately fixed in 10% neutral formaldehyde for 48 h. After embedding in conventional paraffin, HE staining was performed, and the pathologists who were unaware of the grouping were observed by optical microscope and the pathological score was performed. The lung injury score was scored according to the criteria of lung injury pathology of the American Thoracic Society, including: (i) neutrophils in alveolar space; (ii) interstitial space neutrophils; (iii) the number of clear membranes; (iv) the number of protein fragments; And (v) thickening of the alveolar septum, each on a 0–2 scale. The lung injury score is calculated by the following formula: Lung injury score =[20*(i) + 14* (ii) + 7* (iii) + 7* (iv) + 2*(v)]/ 100. Four 400 x field of view scores were selected for each section and averaged. At least 50% of each field of view should be occupied by the alveoli, excluding areas consisting mainly of major airway or vascular lumens [12].

### Determination of IL-6, IL-1β and TNF- α levels in lung tissue

Appropriate amount of lung tissue was taken, lung tissue homogenate was prepared at low temperature, and the contents of IL-6, IL-1β and TNF- α in lung tissue homogenate were detected by commercial ELISA kit (Shanghai Enzyme-Linked Biotechnology Co.,LTD., Shanghai).The ranges of ELISA kit used for IL-1β, IL-6 and TNF-α detecting were 0-120pg/ml, 0-120pg/ml and 0-640pg/ml respectively. The IL-1β and IL-6 ELISA kits demonstrated a sensitivity threshold of 0.1 pg/ml, whereas the TNF-α ELISA kits exhibited a sensitivity of 1 pg/ml.

### Determination of TLR4 and NF-κB in lung tissue

After perfusion, appropriate amount of lung tissue was taken, RIPA lysate was added to ice homogenate, total protein was extracted by grinding instrument at low temperature, centrifuge was centrifuged at 4℃ at 13000r/min for 15 min, and the supernatant was taken with BCA kit (Nanjing Nuoweizan Biotechnology Co.,LTD.,Nanjing) to measure protein concentration. The samples were loaded with 10% SDS-PAGE glue for protein electrophoresis, wet-transferred to PVDF membrane, enclosed with 5% skim milk powder for 1 h, incubated at 4℃ overnight with TLR4(1:1000, CST), NF-κB(1:1000, CST) and β-actin(1:5000, CST) ratio, and washed by TBST for 3 times. The second antibody was incubated at room temperature for 2 h (1:500), and then TBST was washed for 3 times for 10 min each time. ECL supersensitive luminescent reagent was used to avoid light development. Using, β-actin as internal reference, Image J software was used for relative quantitative analysis.

### Statistical methods

Statistical analysis of experimental data was performed using GraphPad Prism 8. Data with normal distribution were expressed as mean ± standard deviation, and statistical analysis of differences between groups was performed by one-way analysis of variance. For data with non-normal distribution, Kruskal-Wallis test was used for multiple comparisons. *P < 0.*05 was considered statistically significant.

## Results

### Protective effect of EA on VILI

**Fig. 2:**
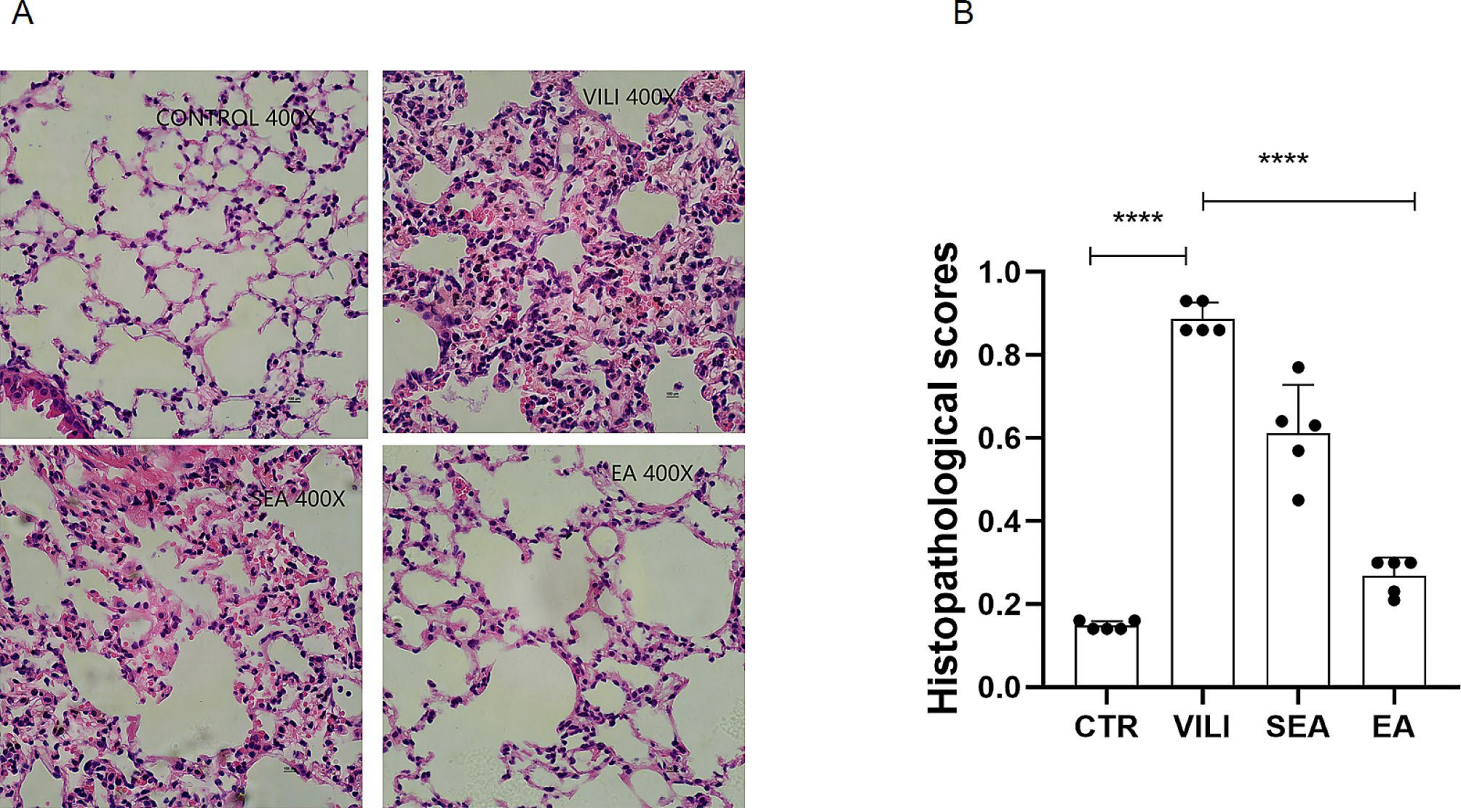
Effect of EA on lung injury associated with mechanical ventilation. **A**: HE staining pathological changes of lung tissues in each group under ×400 sight microscope; **B**: Lung histopathological scores. Data were expressed as mean ± standard deviation, *n* = 5,**P < 0.01. CTR: control group, VILI: ventilator-induced lung injury group, SEA group: sham electroacupuncture group, EA group: electroacupuncture group

The results of lung histopathological morphology showed that the alveolar morphology was normal and the structure of alveolar wall was complete in the CTR group. In VILI group, the alveolar morphology and structure were damaged, the alveolar septum was thickened and a large number of inflammatory cells infiltrated, obvious bleeding and congestion were observed, the alveolar cavity became smaller, and transparent membrane was formed in part of the alveolar. In SEA group, the alveolar septum thickened and a large number of inflammatory cells infiltrated. In EA group, there was no significant thickening of alveolar septum, and a small amount of inflammatory cells infiltrated the pulmonary septum (Fig. 2A). Pathological scores of lung injury showed that the scores of VILI group were higher than those of CTR group, and the scores of SEA and EA groups were lower than those of VILI group, but the differences between EA and VILI groups were statistically significant (*P* < 0.01) (Fig. 2B).

### EA decreased the total protein content and lung tissue water content of BALF

**Fig. 3:**
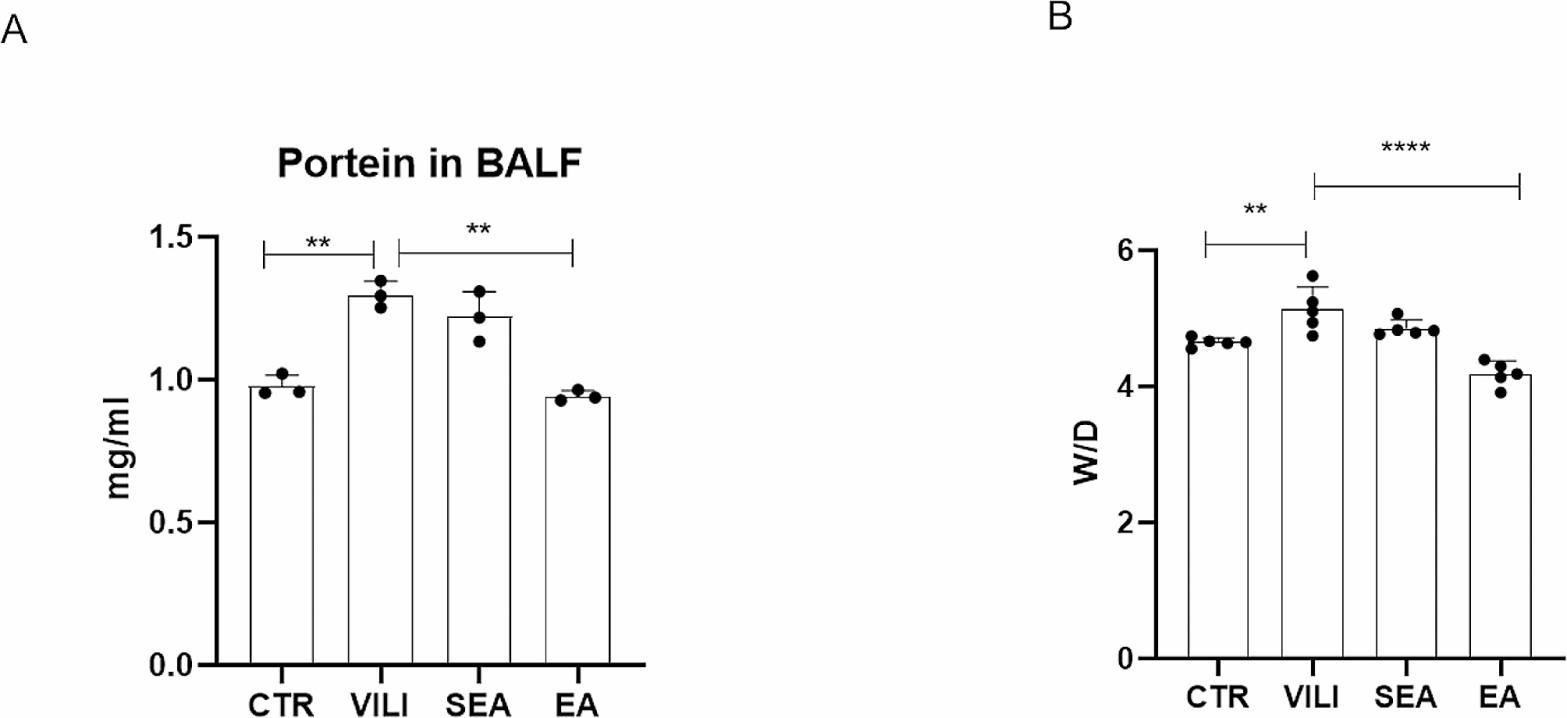
EA reduced the BALF total protein content and water content in the VILI lung. **A**: total BALF protein content, *n* = 3 **B**: wet-dry ratio of lung tissue. Data were expressed as mean ± standard deviation, *n* = 5, **P < 0.01. CTR: control group, VILI: ventilator-induced lung injury group, SEA group: sham electroacupuncture group, EA group: electroacupuncture group

Compared with the CTR group, the total protein content of BALF in the VILI group was significantly increased (*P* < 0.01), while the total protein content of BALF in the EA group was significantly decreased compared with the VILI group (*P* < 0.01) (Fig. 3A). The results of W/D ratio of lung tissue showed that the W/D ratio of VILI group was significantly increased compared with the CTR group. EA treatment could significantly reduce the water content of lung tissue compared with the VILI group (*P* < 0.01) (Fig. 3B). The above results suggest the protective and anti-inflammatory effects of EA on VILI.

### EA reduced the inflammatory factors in the lung tissue

**Fig. 4:**
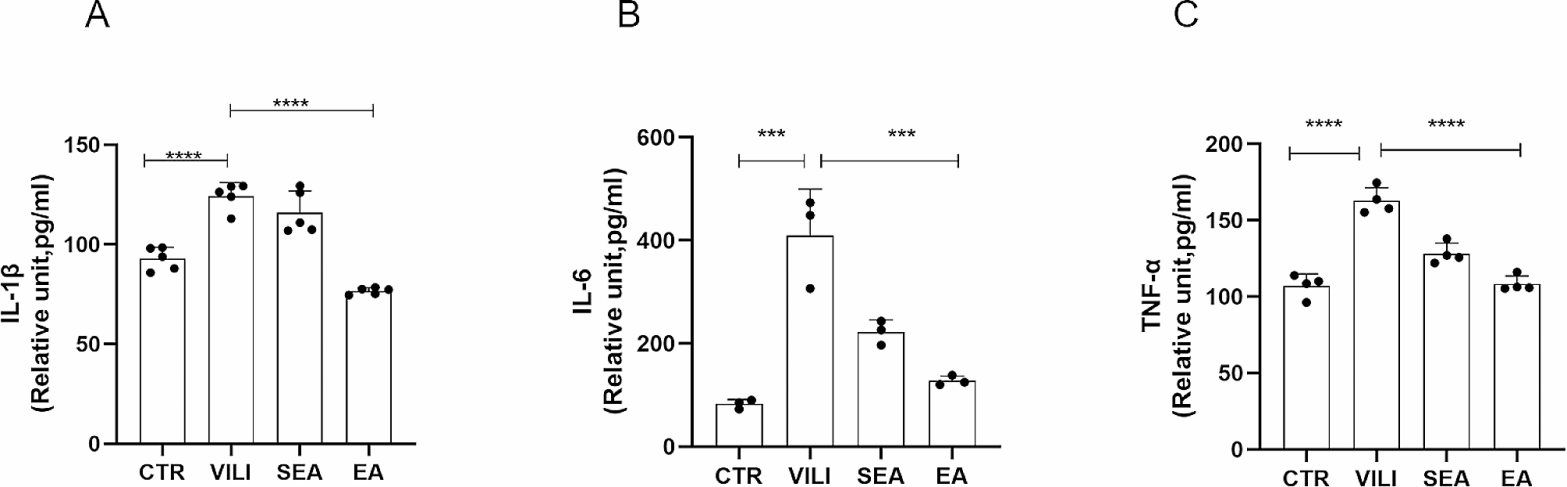
Effect of EA on the content of inflammatory cytokines in VILI lung tissue. (**A**) IL-1β, (**B**) IL-6, (**C**) TNF-α content. Data are expressed as mean ± standard deviation, *n* = 3 or 5, **P < 0.01. CTR: control group, VILI: ventilator-induced lung injury group, SEA group: sham electroacupuncture group, EA group: electroacupuncture group

Compared with the CTR group, the contents of IL-1β, IL-6 and TNF- α in lung tissue of the VILI group mice were significantly increased (*P* < 0.01). Compared with the VILI group, the levels of inflammatory factors IL-1β, IL-6 and TNF-α in lung tissue of EA group mice were significantly decreased (*P* < 0.01) (Fig. 4). However, there was no statistical significance between the SEA group and VILI group.

### EA inhibited TLR4/NF- κB levels in the lung tissue

**Fig. 5:**
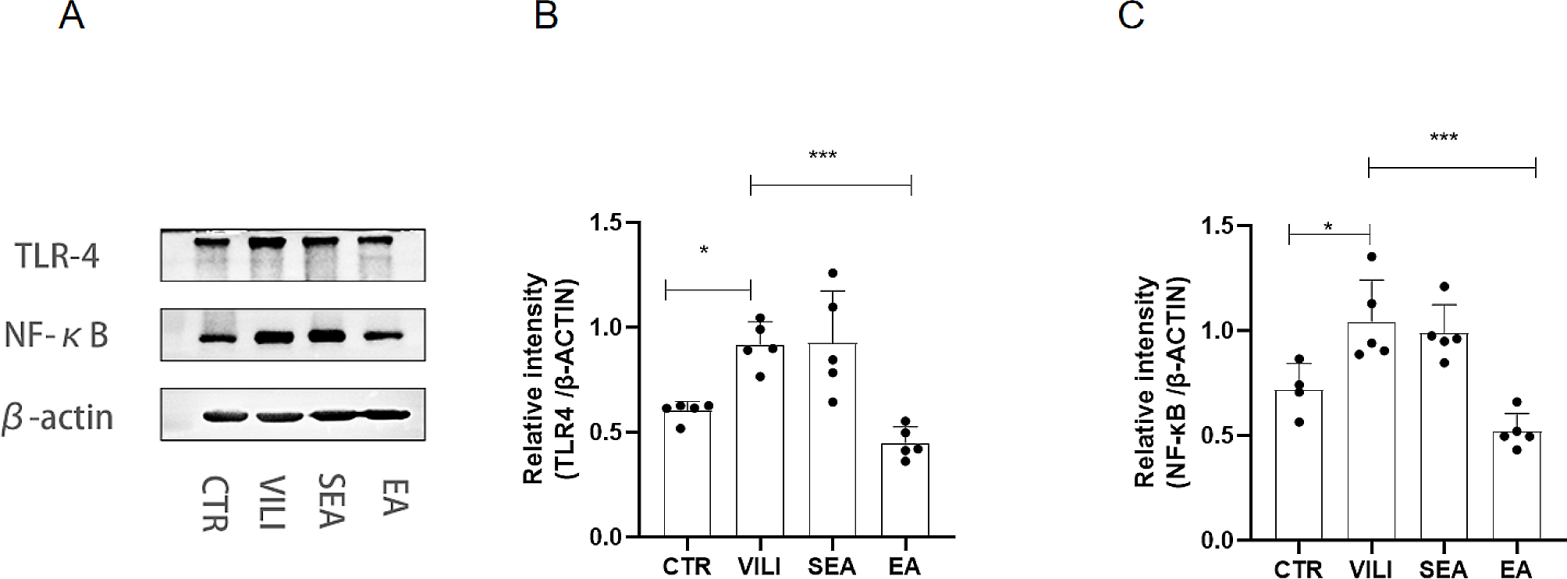
Effect of EA on the relative expression levels of TLR4 and NF-κB. Western blot analysis of (**A**) TLR4 and NF-κB, (**B**) relative expression levels of TLR4 and (**C**) relative expression levels of NF-κB. Results were expressed by mean ± standard deviation (*n* = 5, * *P < 0.*05, **P < 0.*01)*. CTR: control group, VILI: ventilator-induced lung injury group, SEA group: sham electroacupuncture group, EA group: electroacupuncture group

Western blot results showed that the relative expression levels of TLR4 and NF- κB in lung tissue of the VILI group mice were significantly increased compared with CTR group, while EA could significantly decreased the relative expression levels of TLR4 and NF- κB in lung tissue compared with the VILI group (*P* < 0.01) (Fig. 5). However, there was no significant difference in the relative expression levels of TLR4 and NF-κB in lung tissues of SEA group compared with the VILI group.

## Discussion

After 4 h of mechanical ventilation, the lung dry-to-wet ratio, tissue damage score, and bronchoalveolar lavage fluid protein content of ventilator-injured mice were significantly increased. The expression of pro-inflammatory factors (IL-6, IL-1β, TNF-α) and TLR4/NF-κB was also significantly increased.Our study demonstrated that seven days of pretreatment with EA at acupoint ST36 markedly improved pulmonary pathological morphology in VILI mice. This intervention also led to decreased water content and lower expression of inflammatory factors and TLR4/NF-κB in lung tissue.

Ventilator-induced lung injury (VILI) results from mechanical damage that leads to the production and subsequent release of inflammatory mediators in the lung tissue. Research indicates that during mechanical ventilation, the mechanical forces are transduced into biochemical signals by receptors on alveolar epithelial cells, initiating inflammatory signaling pathways within the alveoli. This activation leads to the release of a plethora of inflammatory mediators and cytokines by inflammatory cells, which in turn disrupts the cellular junctions and compromises the integrity of the alveolar membrane. Such events may precipitate pulmonary edema, provoke inflammatory lung damage, and potentially escalate into a systemic inflammatory response syndrome or multiple organ dysfunction syndrome [2, 14]. Our study has revealed that pretreatment with EA at the ST36 over a span of seven consecutive days markedly mitigates lung injury induced by mechanical ventilation. The evidence of this protective effect was primarily observed in the form of reduced pulmonary edema, diminished infiltration and hemorrhage of inflammatory cells, and the attenuation of alveolar membrane thickening.

Acupuncture has a longstanding history of “treating before disease,” which can promote the health of the human body, enhance disease resistance, and prevent the occurrence and progression of illnesses. EA, a combination of traditional acupuncture and modern electrical stimulation, is a simple, stable, and safe method. In 2003, the concept of EA preconditioning was first proposed in an animal experiment about the improvement of nerve damage after cerebral ischemia [15]. Over the ensuing years, scholars from around the globe have delved deeply into the organ-protective mechanisms of electroacupuncture preconditioning. This technique serves as a prophylactic intervention, orchestrating the body’s immune response. Emerging evidence from prior research indicates that mild electrical stimulation of nerves may be beneficial in addressing systemic inflammation, offering a therapeutic pathway for treating inflammatory conditions [16]. A wealth of research has established that a seven-day regimen of electroacupuncture pretreatment can exert potent anti-inflammatory effects across a range of animal models. These models include those for intestinal ischemia-reperfusion injury, myocardial ischemia, intestinal damage, and lung injury induced by sepsis [17–19]. Despite these advances, investigations into electroacupuncture’s protective influence on VILI remain scarce. Our study contributes to this field by revealing that EA can diminish the expression of inflammatory markers in the pulmonary tissues of mice with VILI, thereby displaying significant anti-inflammatory benefits.

Toll-like receptors (TLRs) represent the vanguard of the body’s defense mechanisms against pathogenic incursions. These receptors are adept at recognizing various pathogen-associated molecular patterns (PAMPs) and are pivotal in orchestrating inflammatory responses, as well as in the regulation, survival, and proliferation of immune cells. To date, researchers have identified a total of 11 members within the TLR family. Notably, TLR4 is distinguished as the specific receptor for lipopolysaccharide (LPS), which is a constituent of the outer membrane of Gram-negative bacteria. TLR4 is predominantly expressed in immune cells such as monocytes, dendritic cells, and macrophages. The downstream NF-κB/Rel proteins function as dimerizing transcription factors that regulate gene expression, impacting a myriad of biological processes. These processes encompass innate and adaptive immunity, inflammation, stress responses, B-cell development, and the formation of lymphoid organs [4]. The TLR4/NF-κB pathway is an important pathways modulating the inflammatory response [5], which also plays a very important role in the pathogenesis of VILI [2]. The overexpression of TLR4 triggers intracellular signaling cascades that are associated with pulmonary damage, leading to the enhanced transcriptional activity of NF-κB. Elevated activity of NF-κB is known to compromise cellular and tissue integrity by promoting the release of pro-inflammatory cytokines.

 [20], such as TNF-α and IL-1β. TNF-α is a crucial mediator that can induce apoptosis in lung cells, contributing to tissue injury. It has been documented that the production of TNF-α is an early event in the cascade of various inflammatory conditions, particularly in lung injuries [21]. IL-1β also plays a significant role in both inflammation and immune responses, driving the recruitment of neutrophils and the activation of inflammatory sites [22]. Furthermore, IL-6 is closely linked to inflammatory disorders and is implicated in enhancing NF-κB activation within tissue cells, exacerbating the inflammatory state [23].

Our results suggested that VILI obviously increased the expressions of inflammatory factors IL-1β, IL-6 and TNF-α. While EA could reverse the abnormal elevations of these inflammatory factors as well as reduce TLR4 and NF-κB expression compared with VILI. In summary, the findings of this study propose a potential mechanism by which EA may treat VILI: EA appears to suppress the TLR4/NF-κB signaling cascade, consequently inhibiting the release of downstream pro-inflammatory mediators such as IL-1β, IL-6, and TNF-α, thus mitigating inflammatory damage. Aligned with prior animal and clinical research on sepsis-induced lung injury, EA exhibits an anti-inflammatory action, thereby exerting a protective effect on pulmonary tissues [10].

Our study demonstrated that EA at the ST36 acupoint, when administered as a pretreatment, confers a protective effect against VILI. For clinical patients necessitating mechanical ventilation, early application of EA may serve to reduce lung inflammation attributable to mechanical ventilation and safeguard pulmonary function. This strategy holds promise for reducing the incidence of postoperative pulmonary complications and improving postoperative recovery.

There were limitations in this study. In the interest of avoiding potential tissue damage, TLR4 agonists were not employed to evaluate their capacity to counteract the protective effects of EA on VILI. Moreover, the upstream pathways mediating the anti-inflammatory impact of EA in the context of VILI remain unexplored. Future investigative efforts will be directed towards validating these observations at the cellular level and elucidating the underlying mechanisms responsible for the anti-inflammatory influence of EA.

## Conclusion

Pretreatment with EA at the ST36 acupoint has been shown to ameliorate inflammatory lung injury in a mouse model of VILI though the inhibition of the TLR4/NF-κB pathway. Consequently, the outcomes of this investigation suggest that EA could serve as a viable therapeutic approach for the prevention of VILI.

## Data Availability

The datasets used and/or analysed during the current study available from the corresponding author (yinhong0912@cdutcm.edu.cn) on reasonable request.

## Abbreviations

EA: Electroacupuncture
VILI: Ventilation-induced lung injury
BALF: Bronchoalveolar lavage fluid
MV: Mechanical ventilation
TLRs: Toll-like receptors
PAMPs: Pathogen-associated molecular patterns
TLR4: Toll-like receptors 4
NF- κB: Nuclear factor kappa-light chain enhancer in activated B cells
ARDS: Acute respiratory distress syndrome
CTR: Control
SEA: Sham electroacupuncture
W/D: Wet/dry weight
LPS: Lipopolysaccharide
